# Using Low-Cost Sensors for Fenceline Monitoring to Measure Emissions from Prescribed Fires

**DOI:** 10.3390/s26020745

**Published:** 2026-01-22

**Authors:** Annamarie Guth, Marissa Dauner, Evan R. Coffey, Michael Hannigan

**Affiliations:** Department of Mechanical Engineering, University of Colorado Boulder, 1111 Engineering Drive, Boulder, CO 80309, USA; marissa.dauner@colorado.edu (M.D.); evan.coffey@colorado.edu (E.R.C.); michael.hannigan@colorado.edu (M.H.)

**Keywords:** fencline monitoring, emission factors, prescribed fires, plume detection, low-cost sensors

## Abstract

Prescribed burning is a highly effective way to reduce wildfire risk; however, prescribed fires release harmful pollutants. Quantifying emissions from prescribed fires is valuable for atmospheric modeling and understanding impacts on nearby communities. Emissions are commonly reported as emission factors, which are traditionally calculated cumulatively over an entire combustion event. However, cumulative emission factors do not capture variability in emissions throughout a combustion event. Reliable emission factor calculations require knowledge of the state of the plume, which is unavailable when equipment is deployed for multiple days. In this study, we evaluated two different methods used to detect prescribed fire plumes: the event detection algorithm and a random forest model. Results show that the random forest model outperformed the event detection algorithm, with a detection accuracy of 61% and a 3% false positive rate, compared to 51% accuracy and a 31% false positive rate for the event detection algorithm. Overall, the random forest model provides more robust emission factor calculations and a promising framework for plume detection on future prescribed fires. This work provides a unique approach to fenceline monitoring, as it is one of the only projects to our knowledge using fenceline monitoring to measure emissions from prescribed fire plumes.

## 1. Introduction

As a result of climate change, drier and hotter conditions are causing an increase in wildfire frequency, severity, and intensity [1]. The increase in wildfire activity has contributed to periods of both poor indoor and outdoor air quality [2]. Prescribed fires are an effective way to reduce wildfire risk in wildfire-prone areas [3]. However, prescribed fires release harmful pollutants into the atmosphere, such as particulate matter (PM), carbon monoxide (CO), volatile organic compounds (VOCs), and nitrogen oxides (NOx). Although prescribed fires do emit harmful pollutants, the impact of these emissions is small compared to the total estimated emissions from wildfire [4]. Being able to quantify the emissions from prescribed fire is important for atmospheric modeling of pollutants and for understanding the impact on nearby communities. Additionally, being able to quantify small-scale spatial variability from prescribed fires is important to help inform and improve the inputs to atmospheric emissions models, as well as to understand small-scale variability of emissions, which can disproportionately impact different nearby residents.

This project uses low-cost sensors (LCS) to measure CO, carbon dioxide (CO_2_), and PM_2.5_ from prescribed fires in Colorado and Southeast Georgia. The employed LCS system, called POD, is discussed in more detail in the methods section. These sensor systems are often easy to deploy and thus are a great sampling option for prescribed fire, where the lead time is often only 24–48 h, and the remote nature of the measurements precludes heavy or power-intensive equipment. Due to the relatively low cost of LCS, a larger number of instruments are available, which enables a network of sensor systems for a single combustion event [5,6]. However, LCS are not as accurate and reliable as reference-grade equipment and require frequent calibration [7]. LCS experience signal drift over time, are sensitive to changes in temperature and humidity, and experience cross-sensitivity to other pollutants, meaning they need to be calibrated under the appropriate conditions often to ensure reliable measurements [8,9,10,11,12,13,14]. There have been several studies that have quantified the emissions from prescribed fires [4,15,16,17,18]; however, there has been little work that has measured emissions and quantified emission factors (EFs) using LCS. Using LCS to measure emissions from prescribed fires allows us to measure emissions at several different locations for each burn unit, providing insights into variability, in time and space, of emissions from a singular burn unit.

Fenceline monitoring is the measurement of specific pollutants that cross a known source’s fenceline; in this case, the fenceline would be the burn unit boundaries. The work presented here can be thought of as fenceline monitoring, since emissions are being measured at various boundaries of prescribed fires. Fenceline monitoring is used to detect higher concentrations of target pollutants from known potential sources [19]. Within fenceline monitoring, to the best of our knowledge, there has been no work performed that focuses on detecting prescribed fire plumes. Additionally, there has been no published work focused on detection algorithms for determining whether the emissions are being sampled in the plume of a prescribed fire. To accurately assess emissions using these point-based monitoring PODs, it is vital that we know when a POD is in the smoke plume. EFs (mass of chemical species/mass of fuel burned) were calculated for each burn; however, to undertake this calculation, only the data from the POD while it was in the plume should be used. EFs from each burn are calculated using the partial capture carbon balance method [20]. This method assumes that the samples are from a plume that is well-mixed. Additionally, this method requires background-subtracted values of CO and CO_2_ to be greater than zero in order to calculate a usable EF. So, to calculate an EF from a combustion event, it is important to know when exactly the POD was located in or out of the plume. Here, we present the development and use of an event detection method for the fenceline LCS monitoring system. This paper is organized as follows: Section 2 describes the materials and methods, including the PODs, calibration and harmonization of the PODs, methods to quantify pollutant enhancements and emission factors, POD field deployment, and our development of an event detection algorithm. Section 3 presents the results of POD calibrations, plume detection approaches, and compares the resulting emission factor estimates. Section 4 discusses the implications of the plume detection approaches, the advantages and limitations of using LCS systems for fenceline monitoring, and the broader relevance of these findings for emissions characterization.

## 2. Materials and Methods

Using networks of our LCS systems called PODs, we monitored air pollutant concentrations at 11 different prescribed fires. These prescribed fires took place both in Colorado and Southeast Georgia between October 2023 and September 2024. For the prescribed burns, 5 were pile burns, while the remaining 8 were broadcast burns. Pile burning is a result of mechanical thinning of the forest and removal of biomass [21], while broadcast burning is the burning of land from an acre to thousands of acres [22].

**Figure 1:**
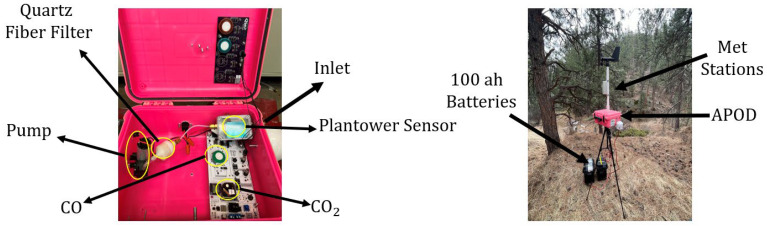
POD layout (on the **left**) and deployed POD setup (on the **right**).

The PODs were developed by the Hannigan Air Quality (HAQ) Lab at CU Boulder and have been used in other emissions measurements as described by Coffey et al. (2017) [23]. The PODs have a variety of different sensors, but here we only report CO, CO_2_, and PM_2.5_ data. CO measurements were collected using the Alphasense CO-B4 sensor (Alphasense, Braintree, Essex, UK), CO_2_ measurements were collected by an ELT-S300 3.3 V sensor (ELT Sensor Corp., Gyeonggido, Bucheon, Republic of Korea), and PM_2.5_ measurements were collected using a Plantower PMS5003 sensor (Nanchang Panteng Technology Co., Nanchang, China). The setup of the PODs is shown in Figure 1 below. The PODs are designed with a pump that pulls air through the inlet, into the casing with the Plantower sensor, through a quartz fiber filter, and then, the air is pulled into the remainder of the POD, where the CO and CO_2_ sensors are located. Additionally, the field setup of a POD is shown on the right panel in Figure 1. The field setup includes a 2 m tall tripod where the POD is attached, 100 amp-hour batteries to power the POD for up to a week, and a meteorological station (MET station) that collects minute-by-minute wind direction and speed data.

### 2.1. Individual Sensor Quantification

#### 2.1.1. CO Quantification

**Figure 2:**
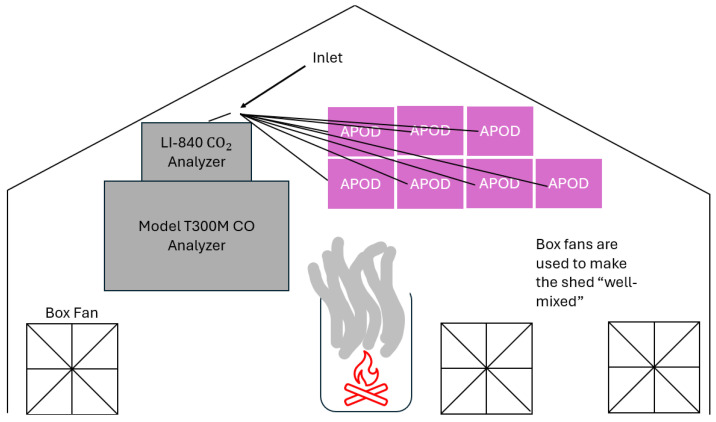
Schematic of the colocation/harmonization setup. Each of the PODs is plumbed to one inlet that is adjacent to both the CO and CO_2_ reference monitors to ensure that the PODs and the reference monitors are sampling the same air. The colocation setup in the shed is the same for both CO, CO_2_, and PM_2.5_. The only difference between the two is that for PM_2.5,_ the reference POD PM_2.5_ concentrations are compared to the remaining PODs, as we do not have a reference-grade PM_2.5_ monitor in the shed.

The POD CO sensor was calibrated using an individual colocation method as described by Okorn and Hannigan [7]. Briefly, a calibrated reference-grade instrument (Model T300M CO analyzer, Teledyne API, San Diego, CA, USA) served as the concentration standard with which we calibrated the sensor signals using a multivariable linear regression; the reference instrument concentration is the dependent variable, and the sensor signals are the independent variables. The reference monitor was calibrated in the lab before being used during a colocation. To calibrate the reference monitor, we employed three gas standards at concentrations of 0, 100, and 450 ppm, which are NIST-traceable. The reference monitor was exposed to each of the gas standards for 15 min to allow the concentration to reach a steady state. Results of the calibration of the CO reference monitor are presented in the Supplemental Information (SI). The POD colocation takes place in a large shed and usually lasts from a few hours to a few days at a time. The PODs are placed next to each other in the shed such that each experiences the same temperature, humidity, and air pollutant concentrations. As the door to the shed can be left open, this shed colocation allows the instruments to experience outdoor temperature and humidity conditions while still being protected from rain and snow. Since the CO sensor calibration includes both temperature and humidity (see Section 2.3.1), to ensure that we are not extrapolating from our calibration parameter space that occurs during our deployments, the ability to reach both high and low temperatures and humidities during calibration is essential. Similarly, to avoid extrapolation during prescribed fire deployments, we needed to generate very high concentrations of CO during the shed colocation. As such, we introduced an emission source into the shed. Specifically, the emission source was either from a gasoline-powered truck or from a small wood fire in a barrel; both of these events produce high concentrations of CO. A photo of the shed colocation POD setup is shown in Figure 2 below. The resulting CO colocation sensor data was cleaned using 3-min medians, while the reference monitor CO concentration data was cleaned using a 3-min average. Medians, instead of averages, are used for PODs to minimize the impact of any short-lived electronic noise that can appear with the POD. In addition to the individual colocation model, we developed a 1-hop colocation model using data collected from one of the colocated PODs. That POD had been colocated with a reference 48i-TLE CO (Thermo Scientific, Waltham, MA, USA) maintained by the Colorado Department of Public Health and Environment (CDPHE) for approximately 6 weeks. The individual colocation in the shed included much higher CO concentrations than the 1-hop colocation at the CDPHE site. Results for the individual CO colocation are discussed in the Results and Discussion sections. Results for the 1-hop CO colocation are presented in the SI. As this method requires more extrapolation for CO concentrations in the field, it was not used for the analysis of field data.

#### 2.1.2. CO_2_ Quantification

CO_2_ was calibrated using an individual colocation method as described by Okorn and Hannigan [7]. As with CO, the sensor signal is calibrated by collocating with a calibrated reference monitor (LI-840 CO2 Analyzer, Lincoln, NE, USA) using a linear regression with the reference monitor CO_2_ serving as the dependent variable and with the CO_2_ raw sensor values, temperature, and humidity serving as the independent variables. The reference monitor was calibrated in the lab before the colocation using NIST-traceable zero air and two span gas standards at concentrations of 1200 and 3200 ppm. The reference monitor was exposed to each standard for 15 min to allow the concentration to stabilize. For results from the calibration of the CO_2_ reference monitor, refer to SI. The colocation took place in a shed as described above for the CO sensor colocation.

### 2.2. 1-Hop Sensor Calibration

#### PM_2.5_ Quantification

For PM_2.5_, a 1-hop colocation method is used as described by Okorn and Hannigan [7]. The 1-hop colocation method uses one POD as the reference POD, which will colocate with a reference monitor for several weeks. This reference POD is then harmonized with the remaining PODs. Harmonization is when the other PODs and the reference PODs are exposed to the same pollutant concentrations, as well as temperature and humidity, and the remaining POD pollutant signals are compared to the reference POD. The POD used as the reference POD in the 1-hop colocation method was colocated for approximately 6 weeks with a Grimm-EDM 180 maintained by CDPHE at a reference site in Denver, CO. For the same reason as explained for CO quantification, the POD PM_2.5_ signal data were cleaned using a 3-min median, while the reference PM monitor data was compiled using a 3-min average. It is important to note that PM concentrations measured at the CDPHE reference site in Denver had a maximum concentration of 50 mg/m^3^, whereas in the field, concentrations reached upwards of 500 mg/m^3^. Due to limited resources, we were unable to add a reference PM_2.5_ monitor to the colocation in the shed; as such, for field PM concentrations that exceed 50 mg/m^3,^ there is added uncertainty as those points are extrapolated. Concentrations of PM_2.5_ at the CDPHE site and concentrations of PM_2.5_ measured in the field are compared in the SI.

### 2.3. Analysis Techniques

**Figure 3:**
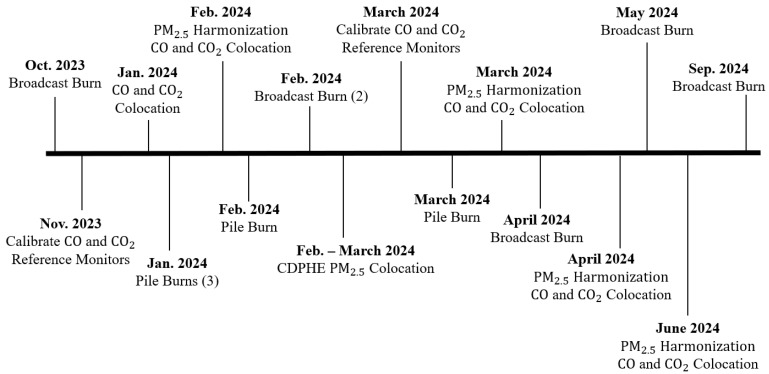
POD colocation and harmonization timeline, reference monitor calibrations, and field data collection. Colocations are completed approximately every 6–8 weeks, and the reference monitors are calibrated every 3–4 months.

An overview of the data collection timeline is presented in Figure 3.

#### 2.3.1. Calibration Analysis

To calculate the concentration of pollutants, the colocation data is split into training and testing data. For testing data, 80% of the colocation data were selected, and the remaining 20% were used for testing. K-fold cross-validation with 3 folds was used, where each fold selected different training and testing data. Each fold randomly selected time chunks of 3-min data.

To calculate the concentrations of pollutants based on the calibration results, the following equation was used.

(1) $$y_{i}=a1+a2\times S_{i}+a3\times humidity+a4\times temperature$$

Equation (1) represents the pollutant concentrations for CO_2_ and PM_2.5_. S_i_ represents the sensor signal at different times, and y_i_ represents the resulting pollutant concentration.

(2) $$y_{i}=a1+a2\times S_{1i}+a3\times S_{2i}+a4\times humidity+a5\times temperature$$

Equation (2) represents the pollutant concentrations for CO. To calculate the resulting CO concentration, the CO sensor produces both a main and auxiliary signal, represented by S_1i_ and S_2i,_ and y_i_ represents the resulting pollutant concentration.

The following metrics were used to assess the performance of the sensor calibrations: R^2^, root mean squared error (RMSE), and the mean bias error (MBE). All analyses were performed in MATLAB 2021b.

(3) $$R^{2}=1-\frac{\sum_{i=1}^{N}{\left(\hat{y_{i}}-y_{i}\right)}^{2}}{\sum_{i=1}^{N}{\left(y_{i}-\bar{y_{i}}\right)}^{2}}$$

(4) $$RMSE=\sqrt{\frac{\sum_{i=1}^{N}{\left(y_{i}-{\hat{y}}_{i}\right)}^{2}}{N}}$$

(5) $$MBE=\frac{1}{N}\sum_{i=1}^{N}{(\hat{y}}_{i}-y_{i})$$

In Equations (3)–(5), y_i_ represents the pollutant concentration measured by the POD and ${\hat{y}}_{i}$ represents the pollutant concentration measured by the reference monitor. N represents the total number of timepoints where pollutant concentration was measured during the colocation period.

#### 2.3.2. Field Data Analysis

The following metrics were used to analyze field data: background-subtracted CO (CO enhancement), background-subtracted CO_2_ (CO_2_ enhancement), background-subtracted PM_2.5_ (PM_2.5_ enhancement), modified combustion efficiency (MCE), and EFs.

(6) $$PE=y_{i}-\bar{y}$$

In Equation (6), PE represents the pollutant enhancement. y_i_ represents the pollutant concentration measured by the POD and $\bar{y}$ represents the average pollutant concentration for the defined background period.

Subtracting background concentrations is important because it allows us to measure what the prescribed burn emissions are adding to the atmosphere, ensuring that we are not using ambient concentrations for EF analysis. Background periods are chosen either before or after the burn when there are no combustion emissions. Typically, the background periods are an average of ambient concentrations over a 5–60-min period. There is a large range in background time periods because, often, in this area of work, we do not have a lot of time before a burn to set up our PODs. For some burns, we obtain a longer time to set up, meaning we have longer background periods. Additionally, for some burns, we set up only minutes before the burn begins, meaning we have to use a background time period after the burn is completed, or if that is not possible, we have to use the small amount of time before the burn begins.

EFs for CO are calculated using two equations detailed below. The first is used to convert the concentration of CO from units of ppm to units of mgm3, where MW is the molecular weight, R is the universal gas constant, T is the temperature, and wt% kg C is the carbon content of the fuel; for this calculation, a value of 50% was used. We chose to use 50% as that is the average carbon content of pine species, which was the dominant fuel type at each prescribed burn used throughout this study [20]. We used the carbon balance method to calculate EFs. This method has been used in other studies as well; both Coffey and coworkers and Roden and coworkers used the same approach and similar carbon content to measure emissions from biomass burning associated with cookstoves [20,23]. Additionally, we calculated the error associated with each EF calculation using Equations (9)–(13) for CO and 14–16 for PM_2.5_.

(7) COmgm3=COppm×Patm×MWCOgmol×103 L1 m3×103 mgg106ppm×RL atmmol K×T(K)

(8) EFgkg−fuel=COmgm3×1 L airCO+CO2 µL×m3103 L air×109 µLkg fuel×1 m3 CO20.4905 Kg C×wt% kgC×10−31 mg

(9) COminmgm3=(COppm−RMSECO)×Patm×MWCOgmol×103 L1 m3×103 mgg106ppm×RL atmmol K×T(K)

(10) COmaxmgm3=(COppm+RMSECO)×Patm×MWCOgmol×103 L1 m3×103 mgg106ppm×RL atmmol K×T(K)

(11) EFmingkg−fuel=COmaxmgm3×1 L airCO+RMSECO+CO2+RMSECO2 µL×m3103 L air×109 µLkg fuel×1 m3 CO20.4905 Kg C×wt% kgC×10−31 mg

(12) EFmaxgkg−fuel=COminmgm3×1 L air(CO−RMSECO)+(CO2−RMSECO2) µL×m3103 L air×109 µLkg fuel×1 m3 CO20.4905 Kg C×wt% kgC×10−31 mg

(13) EFerrorgkg−fuel=EFmax−EFmin×0.5 

Emission factors for PM_2.5_ were calculated using the equation below. MW is the molecular weight, R is the universal gas constant, T is the temperature, and wt% kg C is the carbon content of the fuel; for this calculation, a value of 50% was used.

(14) EFgkg−fuel=PMµgm3×10−3 mgµg×1 L airCO+CO2 µL×m3103 L air×109 µLkg fuel×1 m3 CO20.4905 Kg C×wt% kgC×10−31 mg

(15) EFmingkg−fuel=(PM+RMSEPM)µgm3×10−3 mgµg×1 L airCO+RMSECO+CO2+RMSECO2 µL×m3103 L air×109 µLkg fuel×1 m3 CO20.4905 Kg C×wt% kgC×10−31 mg

(16) EFmaxgkg−fuel=(PM−RMSEPM)µgm3×10−3 mgµg×1 L airCO−RMSECO+(CO2−RMSECO2) µL×m3103 L air×109 µLkg fuel×1 m3 CO20.4905 Kg C×wt% kgC×10−31 mg

(17) EFerrorgkg−fuel=EFmax−EFmin×0.5 

### 2.4. Deployment

Three to six PODs were deployed at each prescribed burn. The number of PODs deployed depended on multiple factors, including the accessibility of the area being burned and the predicted location of the smoke plume during the burn. In some cases, the area being burned was very difficult to access due to large amounts of snow (pile burning) or steep terrain, and as such, PODs were placed in locations that were accessible, which limited the number of locations. Additionally, some burns were happening near private land, which further limited POD placement as they could not be placed on private property. To predict the plume location and inform POD deployment locations, BlueSky Playground, a web-based model developed by the US Forest Service (USFS) [7], was employed less than 24 h prior to a confirmed burn. BlueSky Playground uses HYSPLIT, developed by NOAA, to predict both the plume location and PM concentrations at different points in the plume. Before each burn, a BlueSky Playground model was created by defining the area of the burn as well as employing the predicted weather forecasts in that area. Based on these plume model predictions and the access to the burn unit, a POD placement strategy was developed and implemented.

### 2.5. Plume Detection Method Development

One goal of this paper is to describe the approach used to develop a method which distinguishes POD observations that are based on sampling in the prescribed fire plume from those that were not prescribed. To develop this method, field validation data were collected by having one lab member sit next to the POD and record when they and the POD were in the plume during two different broadcast burns that occurred in the spring and fall of 2024. In total, 30 h of validation data were collected; all the validation hours were during the daytime by one observer. While the validation data was collected, there were three plume states were recorded: (in) plume, no plume, and unsure. A time point was considered to be in the plume when there was visible smoke and a strong smoke smell. Unsure data was when there was a smoke smell, but the plume could not be visually seen. No plume was present when there was neither a visible smoke plume nor a smoke smell. We designed this approach to be able to determine and be very certain of whether a POD was in the plume. We were very conservative in saying a POD was in the plume during the validation.

We explored the use of two different plume detection approaches. The first approach was a custom event detection algorithm written in MATLAB. The second approach was a trained random forest model. For each plume detection approach, the following measurement data were used: PM_2.5_, CO, and CO_2_ enhancements, as well as the change in PM_2.5_ enhancement concentrations between each time step. Simply, the event detection algorithm binned time periods as ‘in plume’ if those enhancements were greater than zero and if the absolute value of PM_2.5_ differences was not zero. All other time periods were binned as ‘no plume.’ If the POD was classified as ‘in plume,’ an EF for that time step was calculated (see Figure S8). The random forest model was run in MATLAB using the function TreeBagger. This function uses an ensemble of bagged decision trees for either classification or regression, which reduces the effects of overfitting in the model. For the random forest model, we used 80% of the validation data as training data and tested the model on the remaining 20% of that data. The model predictors were CO, CO_2_, and PM_2.5_ enhancements, and the change in PM_2.5_ enhancement concentrations between each time step. To select training data, the model would randomly select time chunks of data.

Instead of using the random forest model to predict the uncertain data, that data was left as uncertain in the input to the model. We undertook this strategy because although this would result in less data, this data was more robust, as we are more confident in the time periods that were labeled as ‘in plume’ and ‘no plume.’ One main difference between the event detection algorithm development and the random forest model development was the inclusion of uncertain data. Since the event detection algorithm’s plume decision was made based on whether the enhancements were greater than zero or not, it could not predict the current plume condition as ‘unsure’ like the random forest model.

## 3. Results

### 3.1. Air Sensor Calibration Results

Across all calibrations, the CO sensors across all PODs had an R^2^ between 0.83 and 0.96, RMSEs between 1.8 and 3.5 ppm, and MBEs between −0.01 and 0.02 ppm. Additionally, the range of CO concentrations measured in the field lies within the CO concentrations in the shed colocations (see Figure S5). The CO_2_ sensor calibrations had an R^2^ between 0.77 and 0.97, an RMSE between 12.7 and 30.5 ppm, and an MBE between −0.09 and 0.27 ppm. Based on the results from the CO_2_ sensor calibrations, we decided to remove any EF from the final EF results where the CO_2_ enhancements used in the calculation were less than 30 ppm. Since CO_2_ enhancement is in the denominator, we wanted to ensure that there was a significant enhancement relative to the calibration uncertainty. We chose 30 ppm as that was the upper limit of the RMSE calculated for the POD CO_2_ sensors. For additional detailed results, refer to the SI.

For the primary colocation calibration, the reference POD PM_2.5_ sensor had an R^2^ of 0.822, an RMSE of 3.755$\;µg\times m^{-3}$, and an MBE of −0.0005$\;µg\times m^{-3}$ against the reference-grade instrument. For the harmonization, the PM_2.5_ sensors compared to the reference POD had R^2^s between 0.88 and 0.99, RMSEs between 7.179 and 94.77 $µg\times m^{-3}$, and MBEs between −0.0149–1.01$\;µg\times m^{-3}$. One limitation of the PM_2.5_ sensor calibration is that the PM_2.5_ concentrations measured at the CDPHE site during the primary colocation were lower than those measured in the field. Due to this, we have to extrapolate to calculate higher concentrations of PM_2.5_ in the field. Although this is not ideal, this is the best colocation setup for PM_2.5_ that we have access to. For additional detailed results, refer to the SI.

### 3.2. Event Detection Algorithm

**Figure 4:**
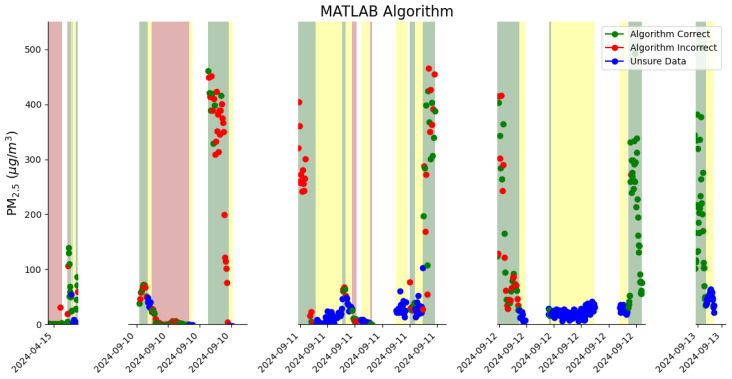
The performance of the event detection algorithm to detect the plume, where shading represents the state of the plume as assessed by the observer, and data points are PM_2.5_ enhancements (using 3-min averaging). The red shading indicates that the POD was not in the plume, and the green shading indicates that the POD was in the plume. The yellow shading is for times with uncertain plume data. Blue data points are shown when the observer was unsure of the plume state. The red dots show where the event detection algorithm predicted the plume state incorrectly. The green dots show where the event detection algorithm predicted the plume state correctly.

Figure 4 shows the performance of the event detection algorithm to classify whether the POD was in the plume. A red dot in green shading indicates that the event detection algorithm did not correctly identify that the POD was ‘in plume’; a red dot in red shading means that the event detection algorithm did not correctly identify that the POD was not in plume. A green dot in red shading indicates that the event detection algorithm correctly identified that the POD was not in the plume, and a green dot in green shading indicates that the event detection algorithm correctly identified that the POD was ‘in plume.’ From looking at this figure, it is noticeable that the PM_2.5_ enhancements increase when the observer records that the POD is in the plume. The event detection algorithm did a much better job at predicting the state of the last two plumes, whereas in the other plumes, the algorithm was not as consistently accurate in predicting the plume state.

### 3.3. Random Forest Plume Detection Model

**Figure 5:**
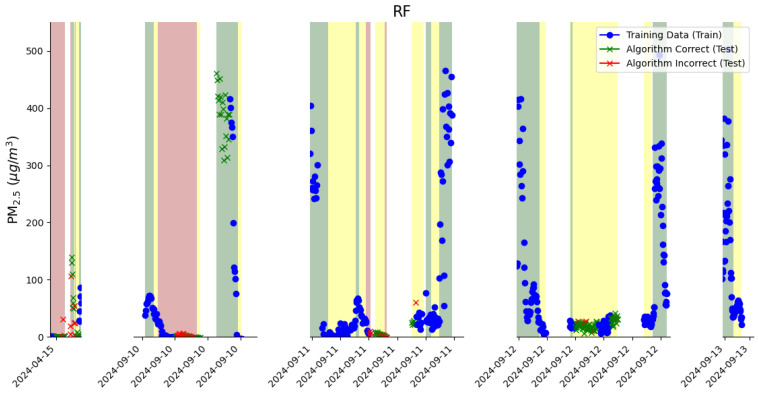
The performance of the random forest model to detect the plume, where shading represents the state of the plume assessed by the observer, and data points are PM_2.5_ enhancements. The red shading indicates that the POD was not in the plume, and the green shading indicates that the POD was in the plume. The yellow shading is for times with uncertain plume data. A green x means that the model was correct, and a red x means that the model was incorrect. Blue points represent the model’s training data.

Figure 5 shows the performance of one run of the random forest model used to detect the plume. Note that each time the random forest model was run, there were different results, as the hours selected for training and testing were chosen randomly. A green x in red shading means the model correctly identified that the POD was not in plume, and a red x in green shading means the model predicted that the POD was not in plume when it was. A green x in yellow shading shows that the model correctly predicted the plume state as unsure. A red x in yellow shading shows that the model incorrectly predicted that the plume state was unsure. The random forest model ranked predictor importance as follows: PM_2.5_ enhancements, CO enhancements, changes in PM_2.5_ enhancements, and CO_2_ enhancements. The final model used an ensemble of 100 decision trees. A sensitivity analysis was performed to determine the optimal number of decision trees to include in the model. These results are discussed in the SI. The random forest model was able to predict whether the plume data is uncertain, meaning there are more robust plume predictions compared to the event detection algorithm, which can only predict ‘in plume’ or ‘no plume.’ In order to determine whether or not to include the ‘unsure’ data in the training data of the random forest model, we did additional model runs where ‘unsure’ data were excluded from the training data and used only in the testing data. This was to assess the differences in resulting EF calculations. However, based on the discrepancy between these results, we determined that the EF calculations were more robust when the random forest model training data included ‘unsure’ data. These results are shown in the SI.

**Figure 6:**
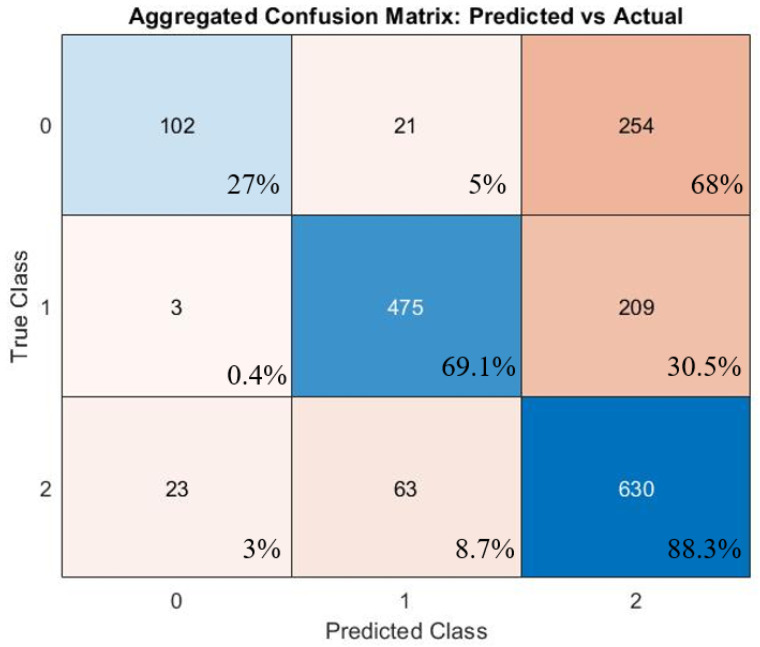
Confusion matrix for the random forest model. In this figure, zero represents ‘no plume,’ one represents ‘in plume,’ and two represents ‘unsure.’ The number in each box represents the frequency of the predicted class compared to the true class for each time point.

Figure 6 is a confusion matrix showing aggregated results across 10 different runs of the random forest model. In this figure, zero represents ‘no plume,’ one represents ‘in plume,’ and two represents ‘unsure.’ Across 10 runs, 5% of the time when an observer assessed ‘no plume,’ the random forest model incorrectly predicted ‘in plume.’ Less than 0.4% of the time, the model incorrectly identified ‘in plume’ as ‘no plume.’ 3% of the time, unsure data was incorrectly identified as not in the plume, whereas 8.7% of the time, unsure data was incorrectly identified as being in the plume. Although the model was not perfect in predicting the plume state correctly, this is more accurate than using the event detection algorithm. The event detection algorithm was correct in predicting the plume 56% of the time, whereas the random forest model was correct in predicting the plume 61% of the time for this specific run. However, the random forest model had false positives 3% of the time, and the event detection algorithm had false positives 31% of the time. So, although the overall accuracy of the random forest model was not much better compared to the event detection algorithm, the random forest model had fewer false positive predictions compared to the event detection algorithm.

### 3.4. Emission Factors

**Figure 7:**
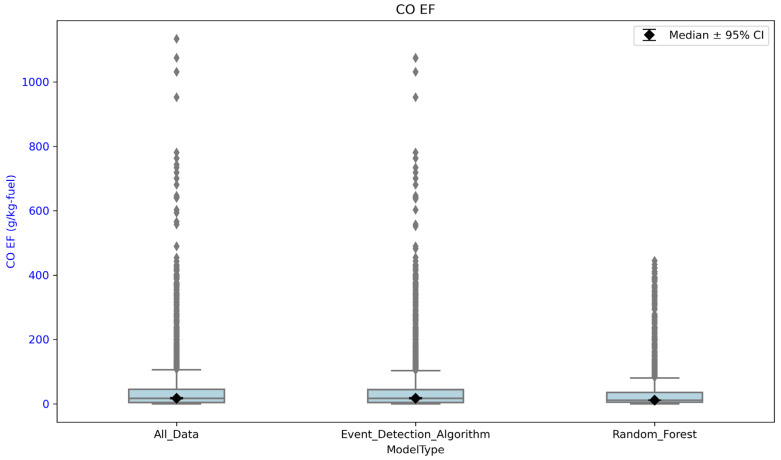
Comparing the EFs for CO across all burns using the event detection algorithm, the random forest model, and no plume detection technique at all.

**Figure 8:**
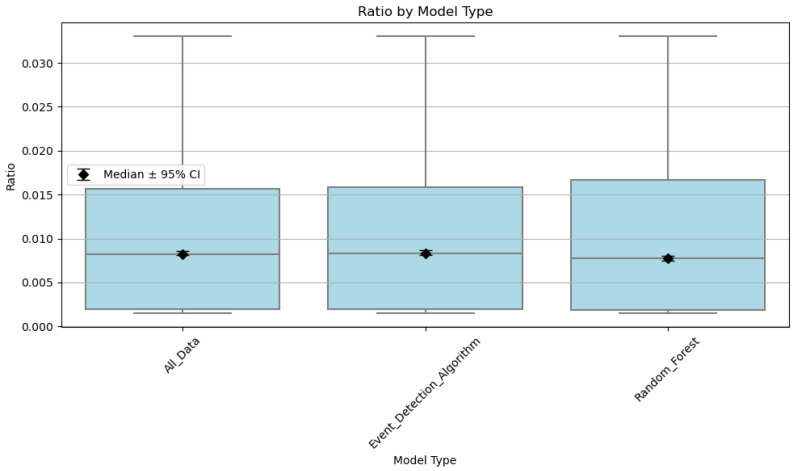
$\frac{1}{\left(CO+{CO}_{2}\right)}$ across 11 prescribed burns.

Figure 7 compares CO EFs across the sampled prescribed burns. Each of the data points is an EF calculated at a time point (the 3-min mean) occurring during each of the prescribed burns. For the event detection algorithm, an EF value is calculated for each time point at which the algorithm detects the POD as ‘in plume.’ For the random forest model, an EF value is calculated for each time point where the model predicted the POD to be ‘in plume.’ When no plume detection method is used (‘All_Data’), an EF value was calculated for each time point during the burn period. Median CO EFs were similar when using the event detection algorithm and when using no plume detection method, 17.36 ± 20.2 and 17.24 ± 20.2 g/kg-fuel, respectively. The CO EFs were lowest when using the random forest model, with a median CO EF of 9.8 ± 20.2 g/kg-fuel. Although the random forest model still had some high CO EFs, there were fewer compared to the event detection algorithm and when not using any event detection method. We compared the mean EFs as well, and there is a statistically significant difference between the mean CO and PM EFs calculated using the random forest model compared to the event detection algorithm using MATLAB. EF values calculated using the random forest model were more similar compared to EFs measured previously in the field by other studies [4,24]. We attribute this to the fact that, when using the random forest model, the concentrations of CO that are very low or zero are not considered as often (which is taken into account when calculating EFs when not using any plume detection or the event detection algorithm). Therefore, higher concentrations of CO are now only being considered for EF calculations, and $\frac{1}{\left(CO+{CO}_{2}\right)}$ is a term in the EF calculation, which would have lower values as there is a division by larger numbers, see Figure 8. This would then result in lower EF estimates when using the random forest model. This is shown below, in Figure 7.

**Figure 9:**
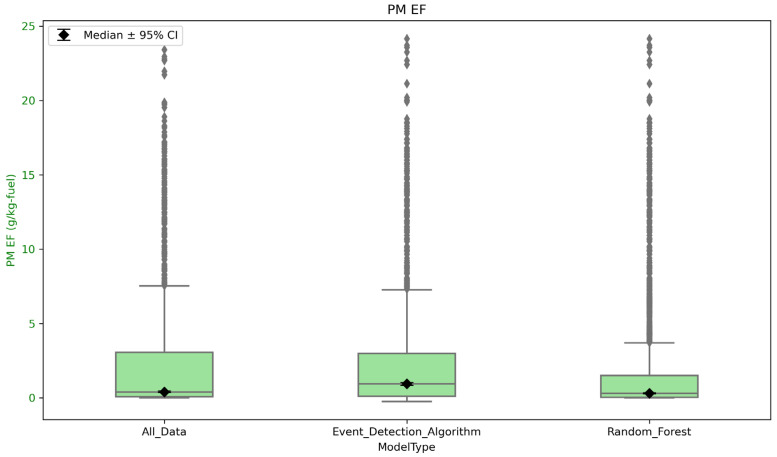
Comparing the EFs for PM_2.5_ across all burns using the event detection algorithm, the random forest model, and no plume detection technique.

Figure 9 compares PM_2.5_ emissions measurements across the same burns as described for Figure 7. Similarly to the CO EFs, the median PM_2.5_ EF when using the random forest model decreased compared to the event detection algorithm but increased compared to when no plume detection method is used. The median PM_2.5_ EF when not using a plume detection method was 0.38 ± 0.04 g/kg-fuel. The median PM_2.5_ EF using the event detection algorithm was 0.92 ± 0.04 g/kg-fuel. The median PM_2.5_ EF for the random forest model was 0.83 ± 0.04 g/kg-fuel. We think this occurred because when we were not using an event detection method, there were points where the PM_2.5_ concentration was zero, but it was still used to calculate an EF. However, when using the event detection algorithm, although time steps where the PM_2.5_ concentration was zero were not used to calculate EFs, very low values of CO were still being used to calculate an EF. This leads to higher median EFs, whereas those low concentrations were excluded from the random forest model.

## 4. Discussion

It is unlikely that the POD is located in the plume for the entire duration of the burn, meaning that cumulative EF calculations can introduce error into the final calculation, as there could be time points where the POD is not located in the plume, but those enhancements are used in the final EF calculation. The results in this work show that there is additional error associated with not using any form of plume detection, and when only using a simple algorithm, the event detection algorithm, to calculate EFs, there is little improvement compared to not using any plume detection method. Overall, the random forest model is a more accurate plume detection method compared to the event detection algorithm, and both are better than not using any plume detection method. The random forest model also has the ability to predict the plume state as ‘unsure,’ meaning that the EFs calculated as a result of the random forest model are more robust. The random forest model also had very few instances where the model predicted that the POD was ‘in plume’ when it was not, and where the model predicted the POD was not in plume when it was ‘in plume,’ meaning it had fewer occurrences of false positives. Additionally, the random forest model had less extreme CO EFs compared to previous studies, as well as compared to the event detection algorithm, and when not using a plume detection method. Additionally, having access to time-series EF data allows access to information on the variability between EFs throughout the entire burn period. EF values can also inform the different phases of combustion and allow us to comment on those different phases, which cannot be performed using a cumulative EF calculation.

These results have broader implications for prescribed fire emissions research and smoke modeling. More accurate plume identification reduces uncertainty in EF estimates, which can lead to improved emissions inventories and air quality monitoring.

## 5. Future Work

Although the random forest model has promising results, future work may be needed to assess the validity of this method across a wider range of fuels and environmental conditions. The random forest model was only developed and validated using 30 h of data from two broadcast burns during the daytime, and all validation data was collected by a single user. This might impact the accuracy of plume characterization at nighttime and might warrant further validation data to be collected at night. Although the random forest model is dependent on pollutant enhancements rather than plume visibility, differences in nighttime smoke emissions were not assessed in this model. Additionally, future work could investigate the use of multiple observers for the collection of validation data. Using only one user introduces potential bias, as determining between ‘in plume’ and ‘unsure’ is subjective. Lastly, it is possible that the random forest model performance could be impacted by the type of fuel being burned. Some fuels have lower carbon contents compared to others, meaning they have the potential to have lower emission intensities, potentially influencing the performance of the random forest model in predicting the plume.

It is important to colocate low-cost air sensors under conditions that are representative of field deployments, where the colocation concentrations are similar to those measured in the field. Future work could involve colocation for PM2.5 under more realistic field conditions to explore if that has any impact on the EF results. This is because our current PM2.5 colocation setup does not reach concentrations similar to what is measured in the field, and, at the CDPHE reference site, the PM2.5 is from vehicle emissions, which have different chemistry compared to PM2.5 from biomass combustion.

Going forward, the random forest model is a promising method to predict whether the POD was ‘in plume’ at prescribed burns.

## 6. Conclusions

Overall, the random forest model represents a promising and robust method for determining plume presence during prescribed burns. Its incorporation into EF calculation workflows can reduce uncertainty and support more accurate characterization of emissions from prescribed fire.

## Data Availability

The data presented in this study is available on request from the corresponding author.

## Supplementary Materials

The following supporting information can be downloaded at: https://www.mdpi.com/article/10.3390/s26020745/s1, Figure S1: CO reference monitor calibration. On the left is the concentration time series for the CO reference monitor; on the right is the voltage-to-concentration equation using the CO reference monitor. Figure S2: CO_2_ reference monitor calibration. On the left is the concentration time series for the CO_2_ reference monitor; on the right is the voltage-to-concentration equation using the CO_2_ reference monitor. Figure S3: Reference PM_2.5_ concentrations versus predicted PM_2.5_ POD concentrations. The dashed 1:1 line indicates that the POD predicted the same concentration as was measured by the reference monitor. For each calibration model, k-fold cross-validation was used with a total of 3 folds. Lastly, ‘cal’ and ‘val’ represent the data that was used as training data and the data that was used as validation data for the model, respectively. Figure S4: Reference CO concentrations versus predicted CO POD concentrations. Figure S5: CDPHE CO concentrations compared to CO concentrations in the field. Figure S6: CO shed colocation compared to CO field concentrations. Figure S7: CDPHE PM_2.5_ colocation compared to PM_2.5_ field concentrations. Figure S8. Simple flow chart for the event detection algorithm. Figure S9: Confusion Matrix for the random forest model predicting unsure values. Figure S10: Comparison between CO emission factors using both the random forest models. ‘Random_Forest’ are the CO emission factors from the model used in the analysis, and ‘Random Forest—No Unsure’ is the new model that uses the random forest to predict the ‘unsure’ data. Figure S11: Comparison between PM_2.5_ EFs using both the random forest models. ‘Random_Forest’ are the CO emission factors from the model used in the analysis, and ‘Random Forest—No Unsure’ is the new model that uses the random forest to predict the ‘unsure’ data. Table S1: $R^{2}$, RMSE, and MBE from the colocation outputs for each POD for CO and CO_2_. Table S2: $R^{2}$, RMSE, and MBE from the colocation outputs for each POD for PM_2.5_. Table S3: $R^{2}$, RMSE, and MBE from the colocation outputs for each POD for CO. Table S4: Sensitivity analysis for random forest models using different numbers of trees.

## Abbreviations

The following abbreviations are used in this manuscript:

CO	Carbon Monoxide
CO_2_	Carbon Dioxide
PM_2.5_	Particulate Matter
LCS	Low-cost Sensors
HAQ Lab	Hannigan Air Quality Lab
EF	Emission Factor
MCE	Modified Combustion Efficiency
