# Supplementary material for: Using Low-Cost Sensors for Fenceline Monitoring to Measure Emissions from Prescribed Fires
Source: Sensors (Basel). 2026 Jan 22;26(2):745. doi: 10.3390/s26020745 (PMC12845575; doi:10.3390/s26020745)
Supplement: Supplementary file 1 [file sensors-26-00745-s001.zip › sensors-4055579-supplementary.pdf]

Supplemental Information

S1. Reference Monitor Calibration

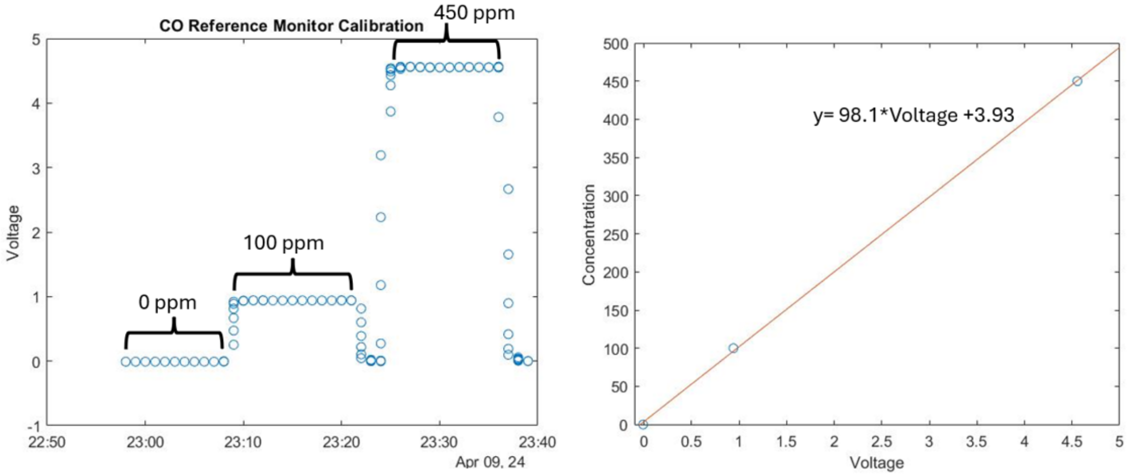

Figure S1. CO reference monitor calibration. On the left is the concentration timeseries for the CO reference monitor, on the right is the voltage to concentration equation using the CO reference monitor.

To calibrate the CO reference monitor, we need to determine which voltages being measured correspond to what concentrations. We use the equation on the right plot to convert voltages recorded from the CO reference monitor in the shed to CO concentrations.

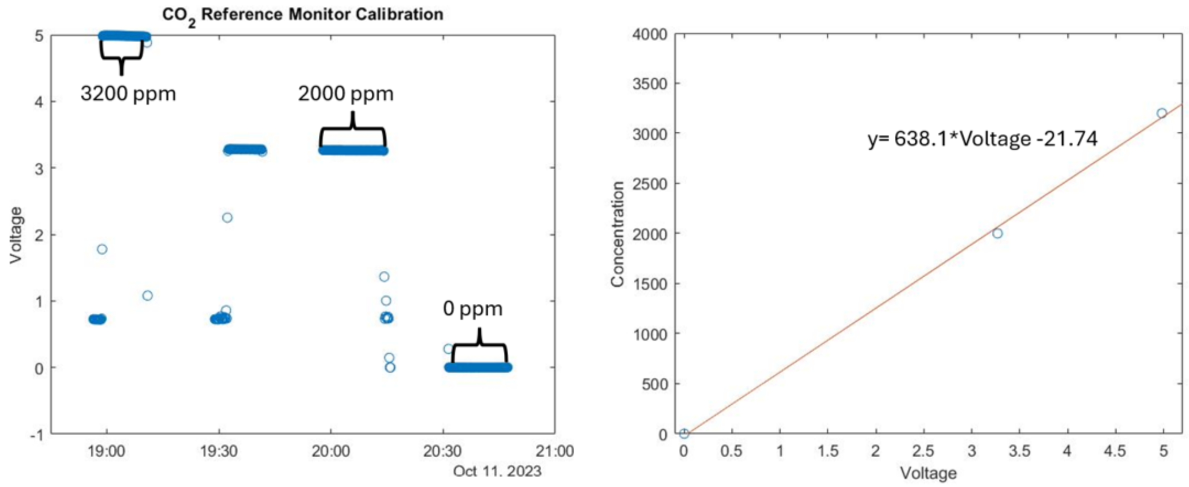

Figure S2. CO_2 reference monitor calibration. On the left is the concentration timeseries for the CO_2 reference monitor, on the right is the voltage to concentration equation using the CO_2 reference monitor.

To calibrate the CO_2 reference monitor, we need to determine which voltages being measured correspond to what concentrations. We use the equation on the right plot to convert voltages recorded from the CO_2 reference monitor in the shed to CO_2 concentrations.

S.2 Individual Sensor Calibration

Table S1. R^2, RMSE, and MBE from the colocation outputs for each POD for CO and CO_2

Pollutant	Colocation Type	R^2	RMSE (ppm)	MBE (ppm)
CO	Individual	0.83 – 0.96	1.8 – 3.5	-0.01 – 0.02
CO_2	Individual	0.77 – 0.97	12.7 - 30.5	-0.09 – 0.27

Table S1 shows the results for the CO and CO_2 sensor colocation. Overall, the CO and CO_2 sensor calibrations for each POD were successful.

S.3 1-Hop Sensor Calibration

Table S2. R^2, RMSE, and MBE from the colocation outputs for each POD for $PM_{2.5}$

Pollutant	Colocation Type	R^2 w/ Ref Monitor	R^2 w/ POD	RMSE w/ Ref Monitor (ug/m^3)	RMSE w/ POD (ug/m^3)	MBE w/ Ref Monitor (ug/m^3)	MBE w/ POD (ug/m^3)
$PM_{2.5}$	1-hop	0.822	0.88 – 0.99	3.755	7.179 – 94.77	-.0005	-.0149 – 1.01

Table S2 shows the results for the $PM_{2.5}$ sensor calibration. Overall, the $PM_{2.5}$ sensor calibration with the reference monitor and for each POD were successful.

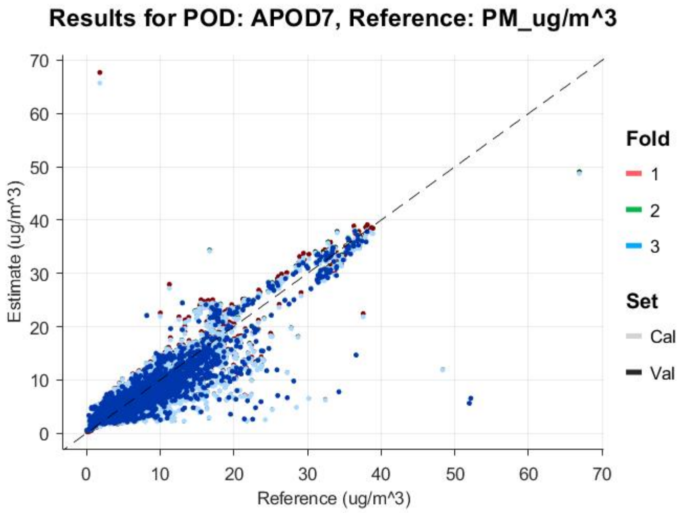

Figure S3. Reference $PM_{2.5}$ concentrations versus predicted $PM_{2.5}$ POD concentrations. The dashed 1:1 line indicates that the POD predicted the same concentration as was measured by the reference monitor. For each calibration model, k-fold cross validation was used with a total of 3 folds. Lastly, "cal" and "val" represent the data that was used as training data and the data that was used as validation data for the model, respectively.

Figure S3 shows the correlation between the reference monitor $PM_{2.5}$ concentrations and A7's, the chosen reference POD, predictions. Overall, the two are highly correlated. The highest $PM_{2.5}$ concentrations were lower compared to what is normally measured in the field.

S4. CO Sensor Calibration using 1-hop Approach

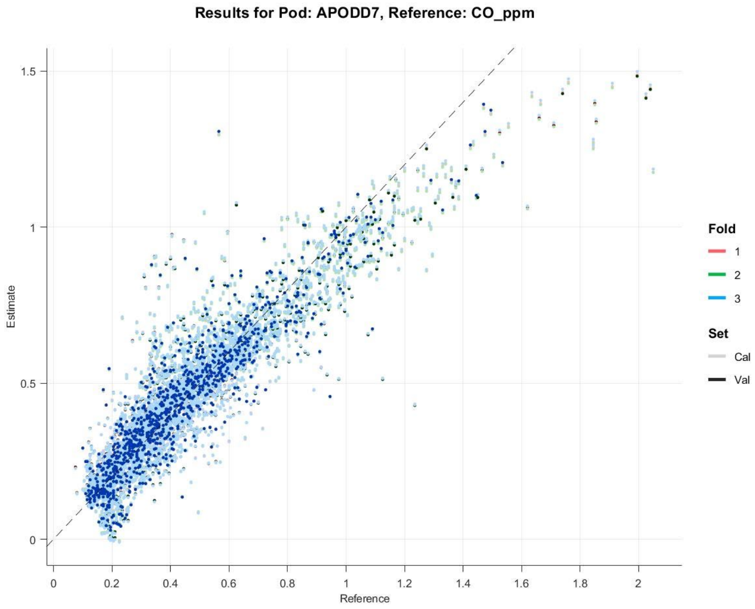

Figure S4. Reference CO concentrations versus predicted CO POD concentrations

Figure S4 shows the correlation between the reference monitor CO concentrations and the POD predictions. Overall, the two are highly correlated. However, the highest CO concentrations were very low compared to what is normally measured in the field (see Figure S5).

Table S3. R^2, RMSE, and MBE from the colocation outputs for each POD for CO

**Pollutant**	**Colocation Type**	**R^2 w/ Ref Monitor**	**R^2 w/ POD**	**RMSE w/ Ref Monitor $\left({}^{ug}/_{m^3}\right)$**	**RMSE w/ POD $\left({}^{ug}/_{m^3}\right)$**	**MBE w/ Ref Monitor**	**MBE w/ POD**
CO	1-hop	0.82	0.93 – 0.99	0.1	0.31 – 0.82	-0.002	-.006 – 0.050

Although the CO 1-hop colocation was successful, the maximum concentrations of the reference POD colocation at the CDPHE site was less than 5ppm, which is not comparative to CO concentrations measured in the field. Because of this, we chose not to use the CO 1-hop in the analysis, as it would result in extrapolation for many of the CO concentrations measured by PODs in the field.

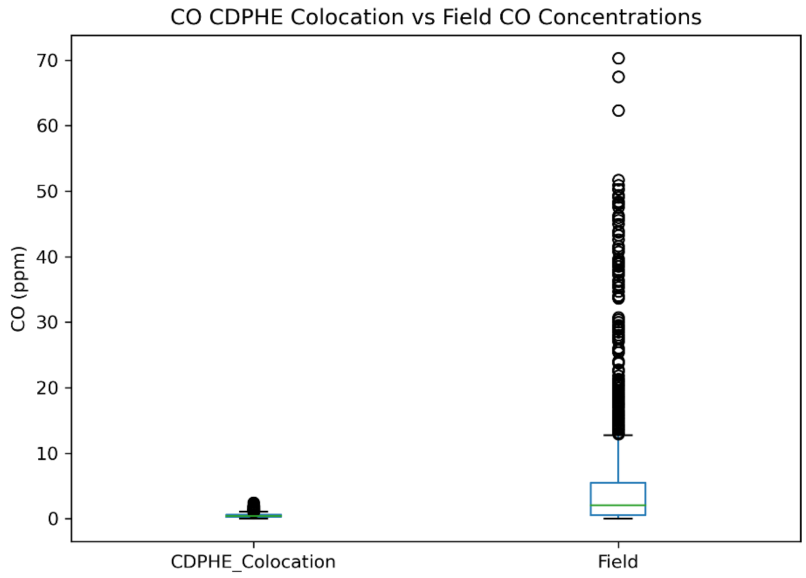

Figure S5. CDPHE CO concentrations compared to CO concentrations in the field

Figure S5 shows the distributions of CO concentrations measured at CDPHE compared to CO concentrations measured in the field. As you can see, the CO concentrations measured at CDPHE are not representative of CO concentrations measured in the field.

S5. Comparison Between Field and Colocation Concentrations

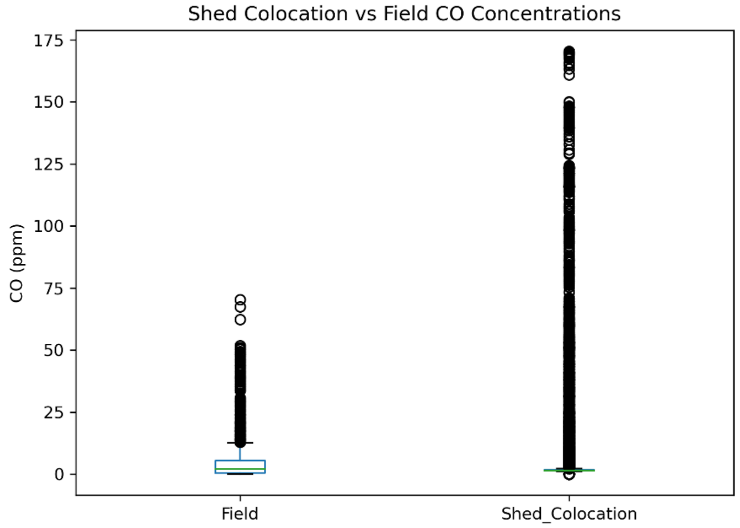

Figure S6. CO shed colocation compared to CO field concentrations

Figure S6 shows the comparison between the CO concentrations measured in the shed during colocation and CO concentrations measured in the field. The CO concentrations in the shed span the same concentrations we measured in the field.

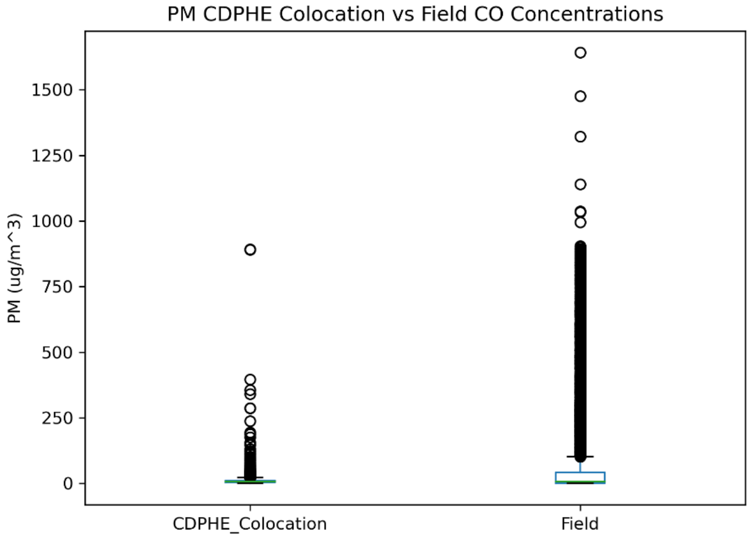

Figure S7. CDPHE $PM_{2.5}$ colocation compared to $PM_{2.5}$ field concentrations

Figure S7 shows the comparison between the $PM_{2.5}$ concentrations measured at CDPHE and in the field. As mentioned previously, the field concentrations exceed the concentrations measured at CDPHE.

S6. Event detection flow chart

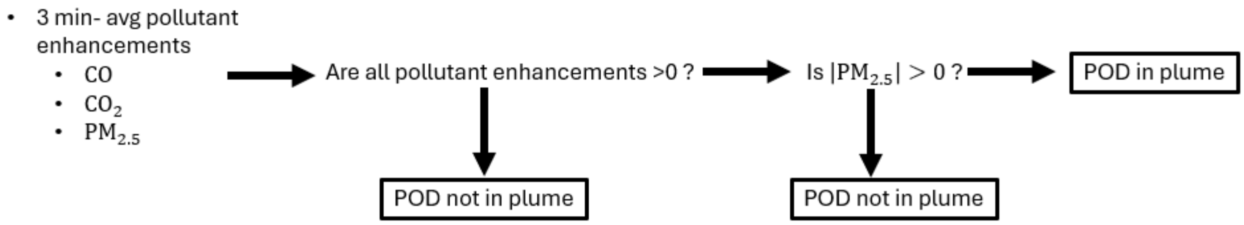

Figure S8. Simple flow chart for the event detection algorithm.

S7. Sensitivity analysis for random forest models

Table S4. Sensitivity analysis for random forest models using different numbers of trees

Number of Trees	False Positive	False Negative
10	14%	2%
25	9%	0%
50	4%	6%
75	5%	22%
100	3%	6%
150	26%	0%
200	17%	0%
250	13%	0%
300	20%	0%
350	49%	5%
400	4%	4%
450	34%	2%
500	21%	13%

Table S4 shows the percentage of both false positive and negative values estimated using the random forest model. A false negative is the random forest model detecting that the POD was not in the plume when it was, and a false positive is the random forest model predicting that the

POD was in the plume when it was not. When selecting the number of trees to use in the analysis, we prioritized the number of trees with the least number of false positives. The model with the lowest false positives was when 100 trees were used, which was the number used in the final analysis.

S8. Using the random forest model to predict 'unsure' plume data

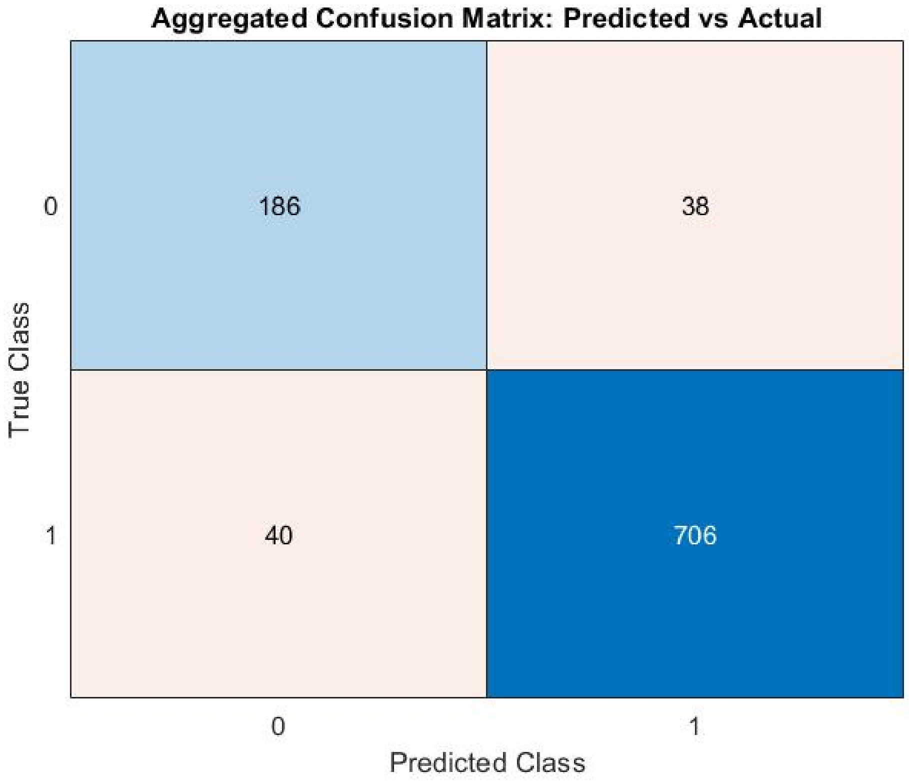

Figure S9. Confusion Matrix for the random forest model predicting unsure values

Figure S9 is a confusion matrix showing aggregated results across 10 different runs of the random forest model. This model uses only data that was in plume or not in plume definitively and does not include 'unsure' data. This model then predicted data that was observed as 'unsure' as either in plume or not in plume. In this figure, 0 represents no plume and 1 represents plume. Across 10 runs, 5% of the time the model incorrectly predicted that a time that was in plume was predicted as not in plume, and 20% of the time, the model predicted 'in plume' when it was not. This model is not as accurate as the one that uses 'unsure' data in the building of the model as it has more false positives.

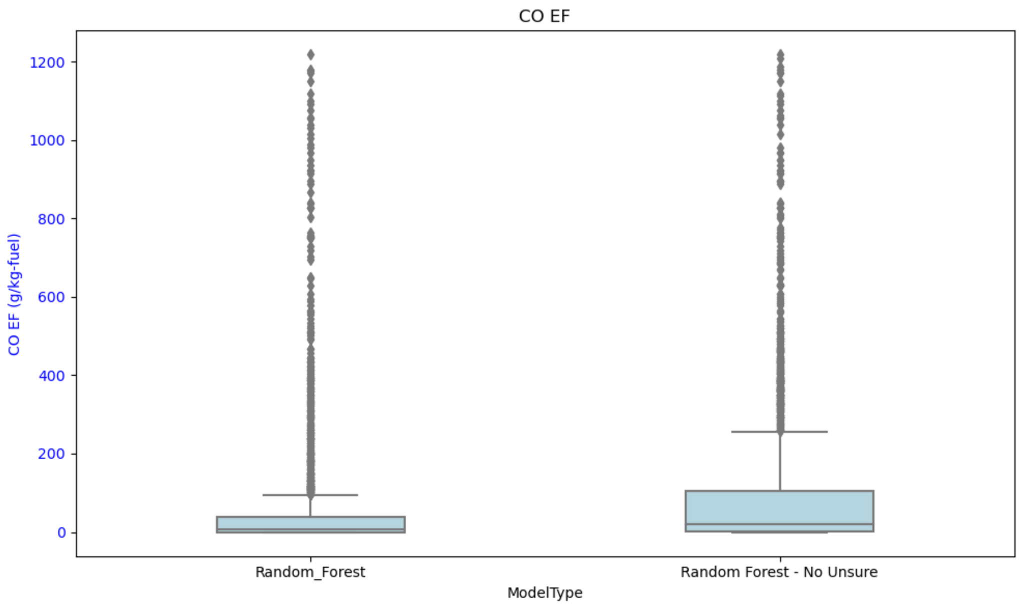

Figure S10. Comparison between CO emission factors using both the random forest models. 'Random_Forest" are the CO emission factors from the model used in the analysis, and 'Random Forest – No Unsure' is the new model that uses the random forest to predict the 'unsure' data.

Figure S10 shows the different CO emission factors using the random forest model that uses 'unsure' plume data to build the model, and the random forest model that does not include 'unsure' plume data in the building of the model. The median CO emission factors in the model not including 'unsure' data is higher.

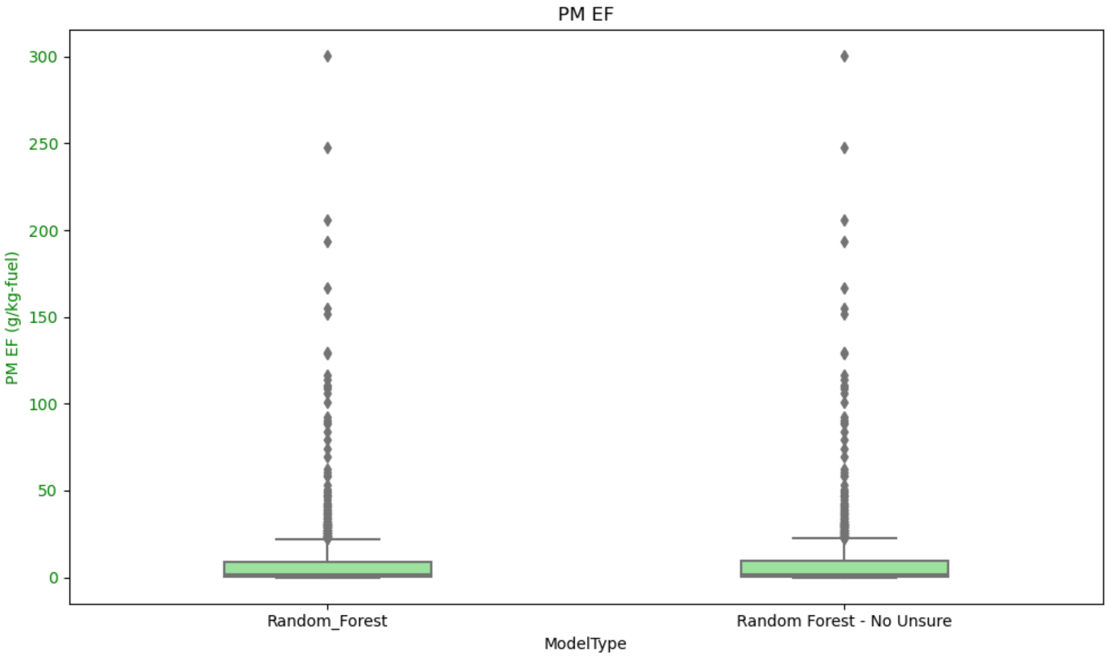

Figure S11. Comparison between $PM_{2.5}$ EFs using both the random forest models. 'Random_Forest" are the CO emission factors from the model used in the analysis, and 'Random Forest – No Unsure' is the new model that uses the random forest to predict the 'unsure' data.

Figure S11 shows the different $PM_{2.5}$ EFs using the random forest model that uses 'unsure' plume data to build the model, and the random forest model that does not include 'unsure' plume data in the building of the model. The median $PM_{2.5}$ EF in the model not including 'unsure' data is very similar to the original random forest model. Although the results for PM are similar, due to the CO EF results, we have decided to use the model that does train on 'unsure' data for our final analysis.
